# Effect of Latanoprost and Timolol–Dorzolamide Therapy on Aqueous Humor Cytokine Concentrations in Patients with Pseudoexfoliative Glaucoma

**DOI:** 10.3390/medicina62091633

**Published:** 2026-08-25

**Authors:** Ivana Valković Antić, Vanda Juranić Lisnić, Vlatka Sotošek, Ivana Bertović, Petra Grubešić, Tea Čaljkušić Mance

**Affiliations:** 1 Clinic of Ophthalomolgy, Clinical Hospital Center Rijeka, Krešimirova 42, 51000 Rijeka, Croatia; ivalkovic@yahoo.com (I.V.A.); grubesic.petra@gmail.com (P.G.); tea.mance.caljkusic@uniri.hr (T.Č.M.); 2 Centre for Proteomics, Faculty of Medicine, University of Rijeka, Braće Branchetta 20, 51000 Rijeka, Croatia; vanda.juranic@uniri.hr (V.J.L.); ibertovic@medri.uniri.hr (I.B.); 3 Department of Anesthesiology, Reanimatology, Emergency and Intensive Care Medicine, Faculty of Medicine, University of Rijeka, Braće Branchetta 20, 51000 Rijeka, Croatia; 4 Department of Clinical Medical Sciences II, Faculty of Health Studies, University of Rijeka, Viktora Cara Emina 2, 51000 Rijeka, Croatia; 5 Department of Ophthalomogy, Faculty of Medicine, University of Rijeka, Braće Branchetta 20, 51000 Rijeka, Croatia; 6 Clinic of Anesthesiology, Intensive Medicine and Pain management, Clinical Hospital Center Rijeka, Vjekoslava Dukića 7, 51000 Rijeka, Croatia

**Keywords:** cytokines, exfoliation syndrome, glaucoma, latanoprost, timolol–dorzolamide

## Abstract

*Background and Objectives*: Pseudoexfoliative glaucoma (PEXG) is a secondary glaucoma caused by the deposition of abnormal flaky protein-like material inside the eye, leading to blockage of the drainage system, increased intraocular pressure (IOP), and optic nerve damage. This study aimed to evaluate the effects of latanoprost and timolol–dorzolamide therapy on the concentrations of interleukin (IL)-1β, IL-6, IL-8, IL-10, IL-12p70, and tumor necrosis factor-alpha (TNF-α) in the aqueous humor and serum samples of patients with PEXG. *Materials and Methods*: This prospective observational study included 60 patients allocated to three age-matched groups (*n* = 20 per group). Group 1 received latanoprost therapy, Group 2 received timolol–dorzolamide therapy, and Group 3 comprised patients with cataracts without PEXG who were not receiving antiglaucoma medication (control group). All participants underwent a comprehensive ophthalmological examination, including visual acuity assessment, specular microscopy, IOP measurement, and intraocular lens (IOL) measurement. Peripheral venous blood was taken from each patient before the surgical procedure and aqueous humor samples were collected during cataract surgery. Concentrations of IL-1β, IL-6, IL-8, IL-10, IL-12p70, and TNF-α were determined using a Cytometric Bead Array and flow cytometry. *Results*: No significant differences were observed in the aqueous humor and serum concentrations of IL-6 and IL-8 between the treatment groups, while IL-1β, IL-10, IL-12p70, and TNF-α did not reach detection. A significant positive correlation was found between IOL and aqueous humor IL-6 concentration in group 1 and between IL-6 and IL-8 serum concentration and IOP in controls. *Conclusions*: Latanoprost and timolol–dorzolamide therapy were not associated with significant differences in aqueous humor or serum IL-6 and IL-8 concentrations in patients with PEXG. IL-1β, IL-10, IL-12p70, and TNF-α were not detectable in the analyzed samples. These findings support the presence of low-grade inflammation in patients with PEXG that does not differ according to the therapy administered. Corneal endothelial alterations are disease-related rather than treatment-induced.

## 1. Introduction

Glaucoma is one of the leading causes of severe visual impairment and blindness worldwide. It is estimated that approximately 76 million people were affected by glaucoma in 2020, with around 4.14 million experiencing moderate-to-severe visual impairment and 3.61 million suffering from blindness [1,2]. Among all forms of glaucoma, primary open-angle glaucoma (POAG) is the most prevalent, accounting for approximately 70% of all cases [3]. It is followed by pseudoexfoliative glaucoma (PEXG), which develops secondary to pseudoexfoliation syndrome (PEXS) [4] and accounts for approximately 25% of all glaucoma cases [5]. The incidence of PEXS increases markedly after the age of 60 years [6,7,8] and is associated with several chronic age-related diseases [9,10,11].

PEXS is characterized by the formation and progressive accumulation of extracellular fibrillar material, observed as gray-white deposits resembling snowflakes [4,12]. This material is deposited on the corneal endothelium, anterior lens surface, iris, ciliary body, and trabecular meshwork, where it creates a mechanical barrier to the normal outflow of aqueous humor [4]. Aqueous humor drains from the anterior chamber primarily through the trabecular outflow pathway located in the iridocorneal angle, accounting for approximately 80–90% of total aqueous humor outflow [13]. Obstruction of the trabecular system by pseudoexfoliative (PEX) material leads to impaired aqueous humor drainage and, consequently, increased intraocular pressure (IOP). Elevated IOP induces structural changes in the optic nerve and functional visual field defects, leading to the development of PEXG [14,15].

Morphological changes initially include increased excavation of the optic nerve head. These changes are followed by functional impairments detectable by visual field testing. PEXG is the most common form of secondary open-angle glaucoma and is typically characterized by more rapid progression, higher IOP levels, a poorer response to medical therapy, and a greater risk of visual loss compared with POAG [4,11]. In the early stages of PEXG, patients are often asymptomatic, and the disease may remain unrecognized or untreated [16]. By the time subjective symptoms appear, the disease is usually advanced; therefore, early diagnosis and timely treatment are essential to prevent irreversible visual damage [16,17]. PEX material may also trigger a local inflammatory response, further damaging the trabecular meshwork and exacerbating resistance to aqueous humor outflow [18]. The pathogenesis of glaucoma involves both mechanical and vascular mechanisms. The mechanical component includes axonal compression resulting from elevated IOP, whereas the vascular component involves reduced blood flow and decreased ocular perfusion pressure in the posterior segment, leading to optic nerve damage [19].

Oxidative stress, induced by factors such as ultraviolet radiation, aging, and nutritional status, plays a significant role in the pathogenesis of PEXS, together with inflammation and ischemia [20,21,22]. Both oxidative stress and inflammation are considered key contributors to the development and progression of PEXG [23,24,25]. Reactive oxygen species generated during oxidative stress may induce cytokine release from inflammatory cells, thereby contributing to extracellular matrix dysregulation in PEXS and PEXG [26].

Cytokines are immune signaling proteins that regulate immune responses and maintain appropriate reactions to infection and tissue injury. However, excessive cytokine production may result in dysregulated immune responses and contribute to the development of inflammatory diseases [27]. Interleukin-8 (IL-8) is a cytokine involved in the recruitment and activation of immune cells. Elevated IL-8 levels may lead to chronic inflammation and disease progression [28]. Its expression is stimulated by other pro-inflammatory cytokines, including IL-1β, IL-6, and tumor necrosis factor-alpha (TNF-α). Studies using a rat model of glaucoma have shown that IL-1β, one of the most potent pro-inflammatory cytokines, contributes to retinal neuroinflammation and retinal ganglion cell degeneration, suggesting a role in the pathogenesis and progression of glaucoma. degeneration, contributing to the pathogenesis and progression of glaucoma [29]. In PEXS, IL-1β is released in response to pathological ocular changes, promoting the activation and recruitment of inflammatory cells [30]. Furthermore, IL-1β alters the metabolism and function of trabecular meshwork cells, increases resistance to aqueous humor outflow, and contributes to elevated IOP [28]. Interleukin-6 (IL-6) is another key pro-inflammatory cytokine that stimulates immune cell activation and proliferation, thereby exacerbating ocular inflammation. It regulates cytokine signaling networks, affects trabecular meshwork function and extracellular matrix metabolism, disrupts aqueous humor drainage, and contributes to increased IOP [31]. In glaucoma, IL-6 is produced by activated glial cells and exhibits both pro-inflammatory and neuroprotective effects, depending on the cellular context and signaling pathway involved [30]. IL-10 is a strong anti-inflammatory cytokine that limits an excessive immune response by inhibiting the activation of helper T cells and reducing the production of pro-inflammatory cytokines, including TNF-α, IL-6, and IL-1β [32]. Additionally, IL-10 reduces oxidative stress and protects retinal ganglion cells from apoptosis. TNF-α is a multifunctional pro-inflammatory cytokine involved in inflammation, cell regulation, and apoptosis. Excessive TNF-α activity can promote retinal ganglion cell injury through the release of toxic mediators and disruption of the retinal microenvironment [30]. IL-12 serves as a critical link between innate and adaptive immunity. It promotes T-helper 1 (Th1)-mediated immune responses by stimulating the production of interferon-gamma (IFN-γ) from T cells and natural killer cells, thereby enhancing cell-mediated immunity [33].

Previous studies have reported abnormal levels of inflammatory mediators in the aqueous humor of glaucoma patients. Increased levels of these factors suggest that inflammation plays a crucial role in the pathogenesis of glaucoma [28,34].

IL-6 is the most extensively studied cytokine; however, findings regarding IL-6 concentrations in PEXG remain controversial. Some studies have reported significantly elevated concentrations in patients with PEXS [26], whereas others have demonstrated significantly decreased levels in POAG and PEXG [35]. Furthermore, aqueous humor concentrations of IL-6 and IL-8 in eyes with late-stage PEXS and PEXG did not differ significantly from those observed in controls [26]. In contrast, concentrations of transforming growth factor-beta (TGF-β) and IL-8 were significantly higher in aqueous humor samples from patients with POAG and PEXG than in those from patients with cataracts [36]. A positive correlation was observed between IOP, the number of glaucoma medications, and increased TGF-β1, IL-8, and SAA levels in the aqueous humor in POAG and PEXG compared with controls [31]. TGF-β is involved in the pathogenesis of PEXG [20,37] and is the only cytokine that has been investigated in relation to antiglaucoma medication therapy. TGF-β regulates cell growth, differentiation, immune responses, and tissue repair by stimulating the production of collagen and fibronectin.

Topical prostaglandin analogs (PGAs) are highly effective in lowering IOP; however, concerns have been raised regarding their potential to disrupt the blood–aqueous barrier and contribute to anterior uveitis or cystoid macular edema (CME), particularly in susceptible patients, such as those with a history of ocular inflammation or recent intraocular surgery [38]. Cases of uveitis and CME have been reported following PGA use, and PGA prescribing information includes warnings regarding intraocular inflammation (uveitis) and CME, particularly in aphakic and pseudophakic patients or in those with known risk factors for macular edema [39]. Nevertheless, no significant differences in the incidence of anterior uveitis or visually significant CME have been observed between PGA-treated and non-PGA-treated patients [40]. Furthermore, glaucoma patients newly initiated on topical glaucoma therapy have not demonstrated an increased risk of uveitis associated with PGA use [41].

To date, the cytokine profile of aqueous humor in patients with PEXG receiving latanoprost or timolol–dorzolamide therapy has not been fully characterized. Therefore, the aim of this study was to investigate the influence of latanoprost and timolol–dorzolamide therapy on concentration of IL-1β, IL-6, IL-8, IL-10, IL-12p70, and TNF-α in the aqueous humor and serum of patients with PEXG.

## 2. Materials and Methods

### 2.1. Patients

This prospective observational study included 60 patients who underwent surgery for cataract in one eye during the period from November 2022 to May 2025 at the Eye Surgery Department at the Ophthalmology clinic, Clinical Hospital Center Rijeka in Rijeka, Croatia.

**Figure 1:**
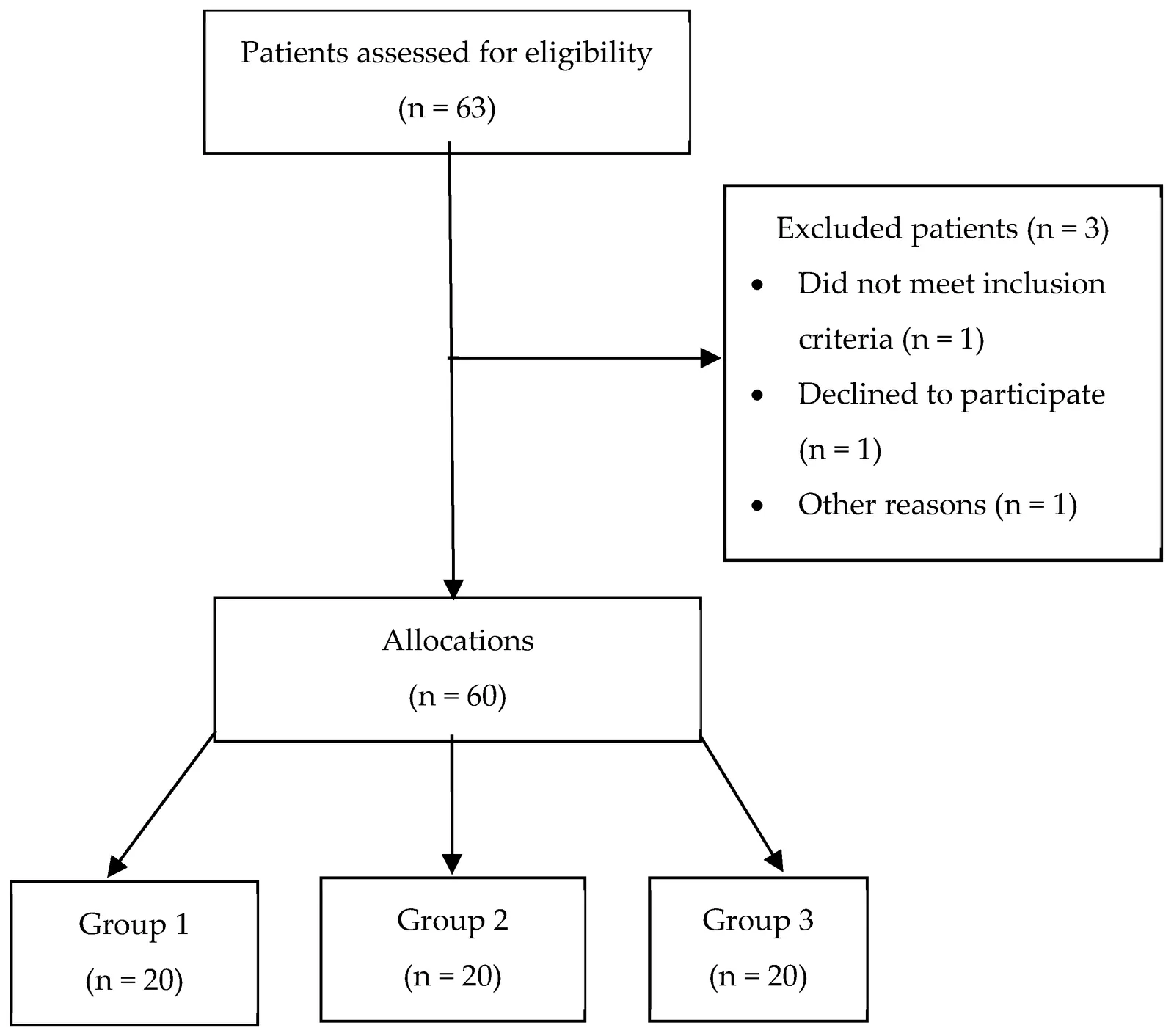
Flow chart of the study protocol.

Considering the medications patients were taking before surgery, the patients were divided into two groups. Group 1 included patients with PEXG and cataract who were receiving the prostaglandin analogue latanoprost, while Group 2 included patients with PEXG and cataract who were receiving the β-blocker timolol maleate and the carbonic anhydrase inhibitor dorzolamide. Each group consisted of 20 patients. The therapy for all patients was not changed for the purposes of the study. The prescribed therapy had been their chronic treatment for at least three months. Patients administered the therapy according to the manufacturer’s instructions. In Group 1, patients instilled the prostaglandin analogue latanoprost (Xalatan, Pfizer, Pfizer Manufacturing Belgium NV, Puurs-Sint-Amands, Belgium) once daily. In Group 2, patients received the β-blocker timolol maleate (Timalen, Jadran-galenski laboratorij, Rijeka, Croatia) at a concentration of 5 mg/mL and the carbonic anhydrase inhibitor dorzolamide (Trusopt, Santen Oy, Tampere, Finland) at a concentration of 20 mg/mL, both twice daily. Group 3 included patients with cataracts who did not have PEXG and were not receiving antiglaucoma therapy. The control group consisted of 20 patients (Figure 1).

Before the surgical procedure, patients were informed about the study protocol and invited to participate. Written informed consent was obtained from all participants. The protocol was approved by the Ethics Committee of the Clinical Hospital Centre Rijeka and the Committee of the Faculty of Medicine Rijeka, University of Rijeka, Rijeka, Croatia (003-05/22-1/31; 2170-29-02/1-22-2) and was conducted according to the World Medical Association criteria in the Declaration of Helsinki.

Patients with a history of previous eye trauma, diabetes, intraocular or systemic infection, immunological disease, laser photocoagulation, cryotherapy, or intraocular surgery were excluded from the study.

### 2.2. Assessment of Visual Acuity, Intraocular Pressure, Central Corneal Thickness, Endothelial Cell Density, and Intraocular Lens Power

For each patient included in the study, visual acuity (VA), IOP, central corneal thickness (CCT), endothelial cell density (ECD), and intraocular lens (IOL) power were assessed. VA was measured by trial frame using an optotype chart, IOP was measured by applanation tonometry, CCT and ECD by specular microscopy (Specular Microscope EM-3000, Tomey Corporation, Nagoya, Japan) and IOL by IOL master (IOLMaster 700, Jena, Germany).

### 2.3. Aqueous Humor and Peripheral Blood Sample Collection

Aqueous humor samples from all patients included in the study were collected at the beginning of surgery. After preparation of the surgical field and administration of local anesthesia with lidocaine hydrochloride (Xylocain 2% gel, Aspen Pharma Trading Limited, Dublin, Ireland), a side-port incision was made using a 15° blade. A total of 50 µL of aqueous humor was aspirated from the anterior chamber using a 27-gauge needle attached to a 1 mL syringe. The samples were then transferred into sterile tubes and stored at −80 °C until further analysis.

Five mL of peripheral venous blood was taken from each patient before the surgical procedure. The samples were stored in test tubes, transported to the central laboratory of the Clinical Hospital Center Rijeka, centrifuged, and the resulting serum was stored at −80 °C until analysis.

### 2.4. Cytokine Measurements in Aqueous Humor and Serum

Quantification of IL-1β, IL-6, IL-8, IL-10, IL-12p70 and TNF concentrations in aqueous humor and serum was performed using a cytometric bead array (CBA) method with a commercially available human inflammatory cytokine kit (BD Biosciences, Heidelberg, Germany), Catalog No. 551811, according to the manufacturer’s instructions. The kit contained six distinct bead populations with varying allophycocyanin (APC) fluorescence intensities, each conjugated with a specific antibody targeting an individual cytokine. Each bead bound its corresponding cytokine, after which a phycoerythrin (PE)-conjugated detection reagent was added to enable signal quantification.

Cytokine standards were prepared by serial dilution (1:2 to 1:256) following the manufacturer’s protocol to generate standard curves for quantitative analysis. Bead suspensions for individual cytokines were briefly vortexed and combined into a single working solution.

For each sample, 50 µL of the bead mixture and 50 µL of aqueous humor were added to the tube. Samples were incubated for 1 h and 30 min at room temperature in the dark. Subsequently, 1 mL of wash buffer was added, and samples were centrifuged at 200× *g* for 5 min, after which the supernatant was carefully removed. Next, 50 µL of PE-conjugated detection reagent was added, and samples were incubated for 1 h and 30 min under the same conditions. After incubation, samples were washed and resuspended in 300 µL of wash buffer. Cytokine concentrations were analyzed using a flow cytometer (FACSAria II, BD Biosciences, Heidelberg, Germany).

### 2.5. Flow Cytometry Acquisition and Analysis

Prepared samples for cytokine concentration analysis were measured using a FACSAria II flow cytometer (BD Biosciences, San Jose, CA, USA) according to standard operating procedures. Beads were detected in the APC channel to distinguish populations with different fluorescence intensities, while PE fluorescence was used to quantify bound detection antibodies. A minimum of 5000 beads per sample was acquired and analyzed.

The resulting data were processed using FlowJo software v. 10 (BD Biosciences, Heidelberg, Germany). Cytokine concentrations were determined by interpolation from the corresponding standard curves and expressed in pg/mL. For each cytokine, median fluorescence intensity and standard deviation were calculated. Samples with values outside the assay detection range were reported as below or above the limits of quantification.

### 2.6. Statistical Analysis

Statistical processing of the collected data was performed using a personal computer. A database was created in MS Excel (Microsoft, Redmond, USA), and data processing and analysis were conducted using the statistical software package TIBCO Statistica 14.1.0.8 (TIBCO, Tulsa, OK, USA). Sample size estimation was performed using G*Power software (version 3.1.9.7; Heinrich Heine University Düsseldorf, Düsseldorf, Germany). Assuming a two-sided significance level (α) of 0.05 and a statistical power of 80% (β = 0.20), the required sample size was calculated to be 20 participants per group. Accordingly, the study was designed to include three groups with at least 20 participants each. Values of categorical variables were described by frequency or percentage. The normality of the distribution of continuous numerical data was tested using the Kolmogorov–Smirnov test. The data showed non-normal distribution; the differences between groups were evaluated with the Kruskal–Wallis test, followed by the Mann–Whitney U-test for post hoc comparisons, with Bonferroni correction applied for multiple testing and adjusted significance threshold of *p* < 0.025.

Correlations were assessed using Spearman’s rank correlation coefficient. Categorical variables were analyzed with the chi-square test or Fisher’s exact test, as appropriate and a *p*-value < 0.05 was considered statistically significant. Data are presented as 25th–75th percentile values.

## 3. Results

### 3.1. Demographic and Clinical Data of the Patients

The demographic and clinical characteristics of patients in Group 1, Group 2, and Group 3 are presented in Table 1. No significant differences in age were observed among the groups. Women accounted for 53.3% of the study population; however, the sex distribution differed significantly between the groups. Specifically, Group 2 had a statistically significant higher proportion of female patients in comparison to Group 1 (*p* = 0.002).

Comparisons of VA, IOP, and ECD among Group 1, Group 2, and Group 3 revealed no statistically significant differences. In contrast, CCT differed significantly among the groups. Group 1 exhibited statistically significant lower CCT values compared with both Group 2 and Group 3 (Group 1 vs. Group 2, *p* = 0.020; Group 1 vs. Group 3, *p* = 0.019). The implanted IOL power also differed significantly among the groups (*p* = 0.015), with statistically significant higher values observed in Group 2 than in Group 3 (*p* = 0.03).

### 3.2. The Influence of Latanoprost and Timolol–Dorzolamide Therapy on Cytokine Concentration in Aqueous Humor

**Figure 2:**
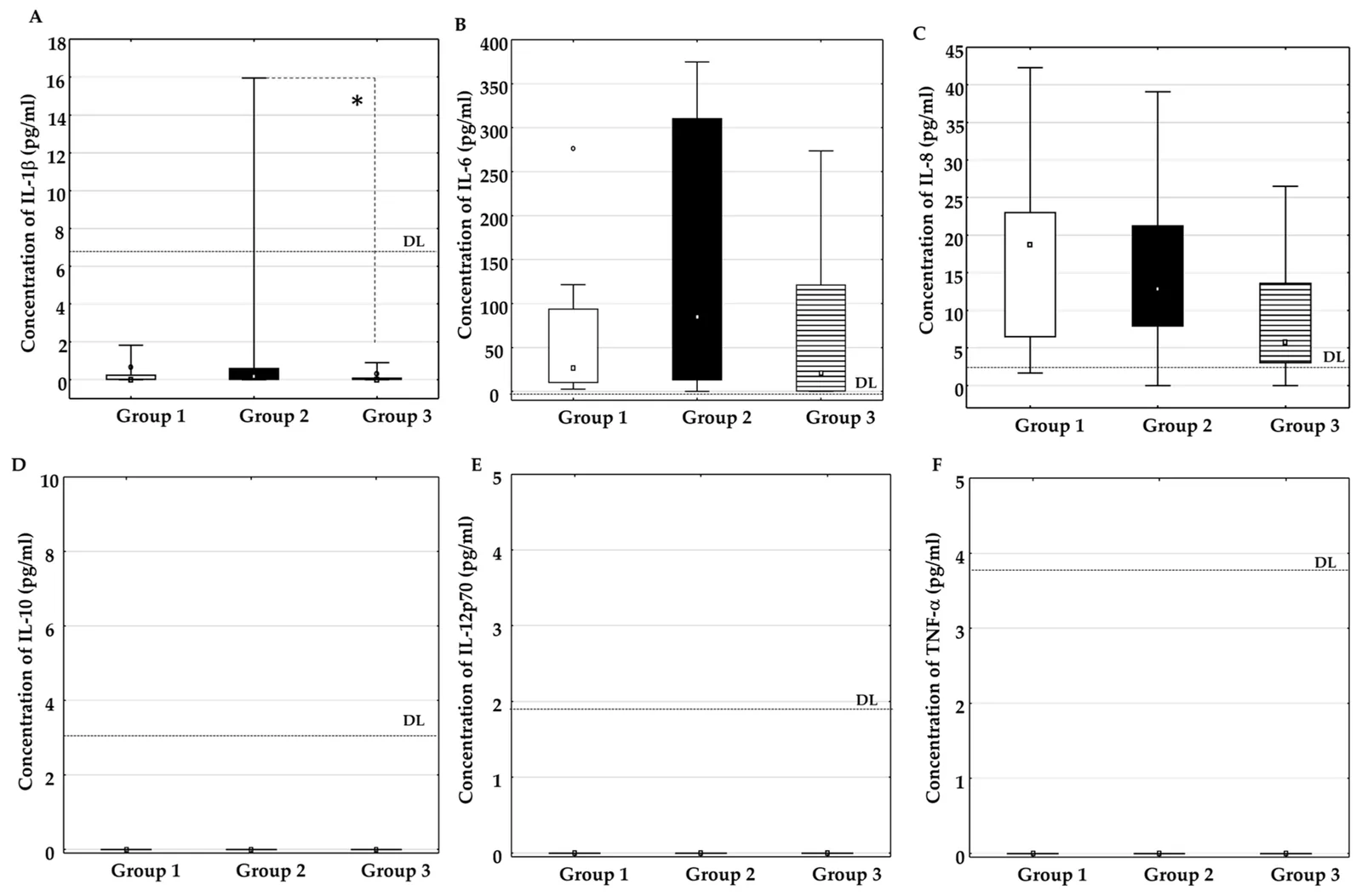
Comparison of IL-1β (**A**), IL-6 (**B**), IL-8 (**C**), IL-10 (**D**), IL-12p70 (**E**), and TNF-α (**F**) in aqueous humor among Group 1 (□), Group 2 (■) and Group 3 (=). * Level of statistical significance: *p* < 0.025. Data are expressed as median and 25th–75th percentile. DL—detection limit.

The influence of latanoprost and timolol–dorzolamide therapy on the concentrations of IL-1β (Figure 2A), IL-6 (Figure 2B), IL-8 (Figure 2C), IL-10 (Figure 2D), IL-12p70 (Figure 2E), and TNF-α (Figure 2F) in the aqueous humor was assessed. The concentrations of IL-1β, IL-10, IL-12p70, and TNF-α were below the assay detection limit. IL-6 and IL-8 were detectable; however, no statistically significant differences in their concentrations were observed among the experimental groups.

### 3.3. The Influence of Latanoprost and Timolol–Dorzolamide Therapy on Cytokine Concentration in Serum

**Figure 3:**
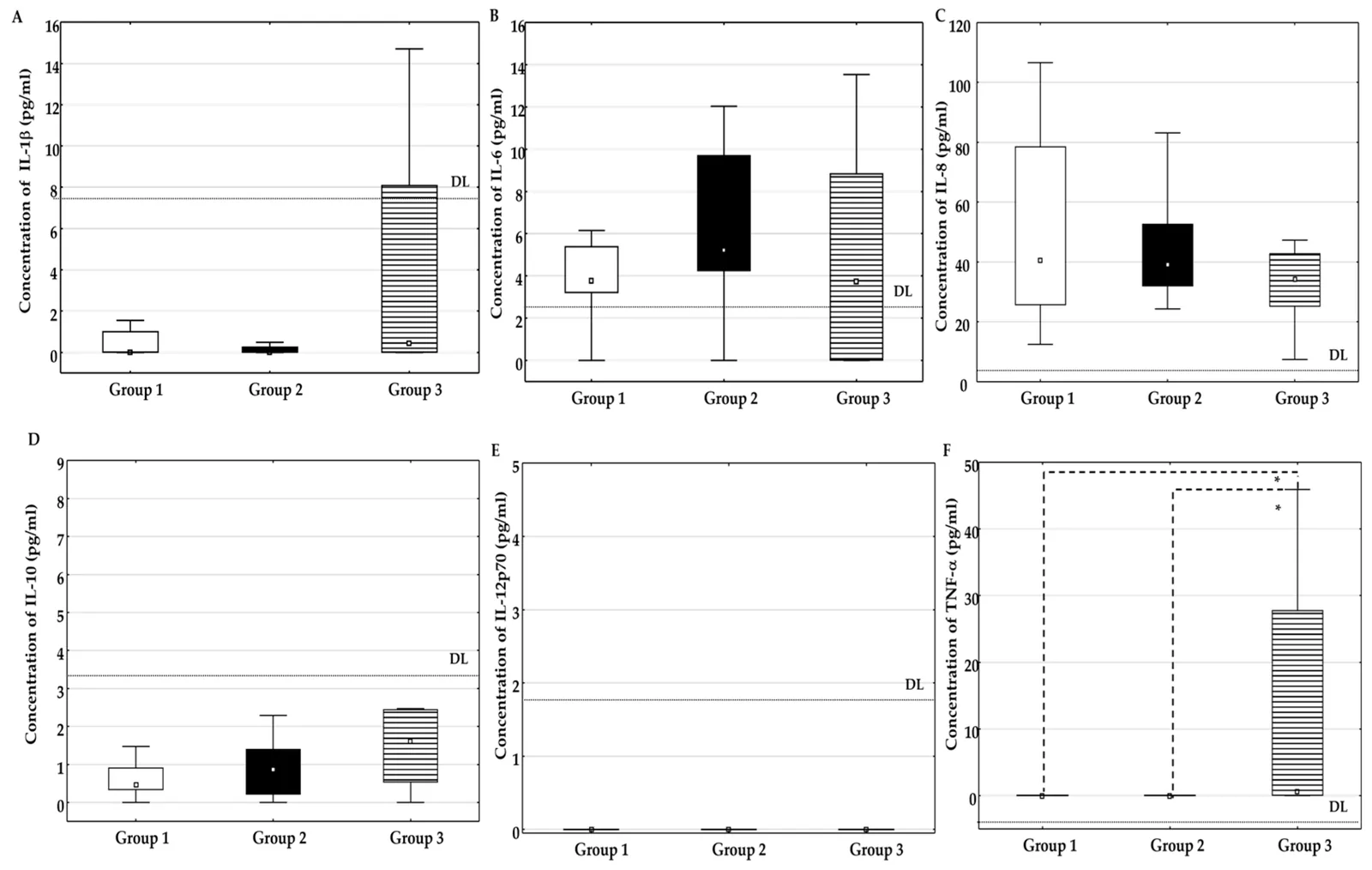
Comparison of IL-1β (**A**), IL-6 (**B**), IL-8 (**C**), IL-10 (**D**), IL-12p70 (**E**), and TNF-α (**F**) in serum among Group 1 (□), Group 2 (■) and Group 3 (=). * Level of statistical significance: *p* < 0.025. Data are expressed as median and 25th–75th percentile. DL—detection limit.

The influence of latanoprost and timolol–dorzolamide therapy on the concentrations of IL-1β (Figure 3A), IL-6 (Figure 3B), IL-8 (Figure 3C), IL-10 (Figure 3D), IL-12p70 (Figure 3E), and TNF-α (Figure 3F) in serum was assessed. The concentrations of IL-1β, IL-10 and IL-12p70 were below the assay detection limit. IL-6, IL-8 and TNF-α were detectable. No statistically significant differences in the concentrations of IL-6 and IL-8 were observed among the experimental groups, but concentration of TNF-α was statistically higher in Group 3 when compared to Group 1 and Group 2.

### 3.4. Comparison Between Cytokine IL-6 and IL-8 Concentrations in Aqueous Humor and Serum

**Figure 4:**
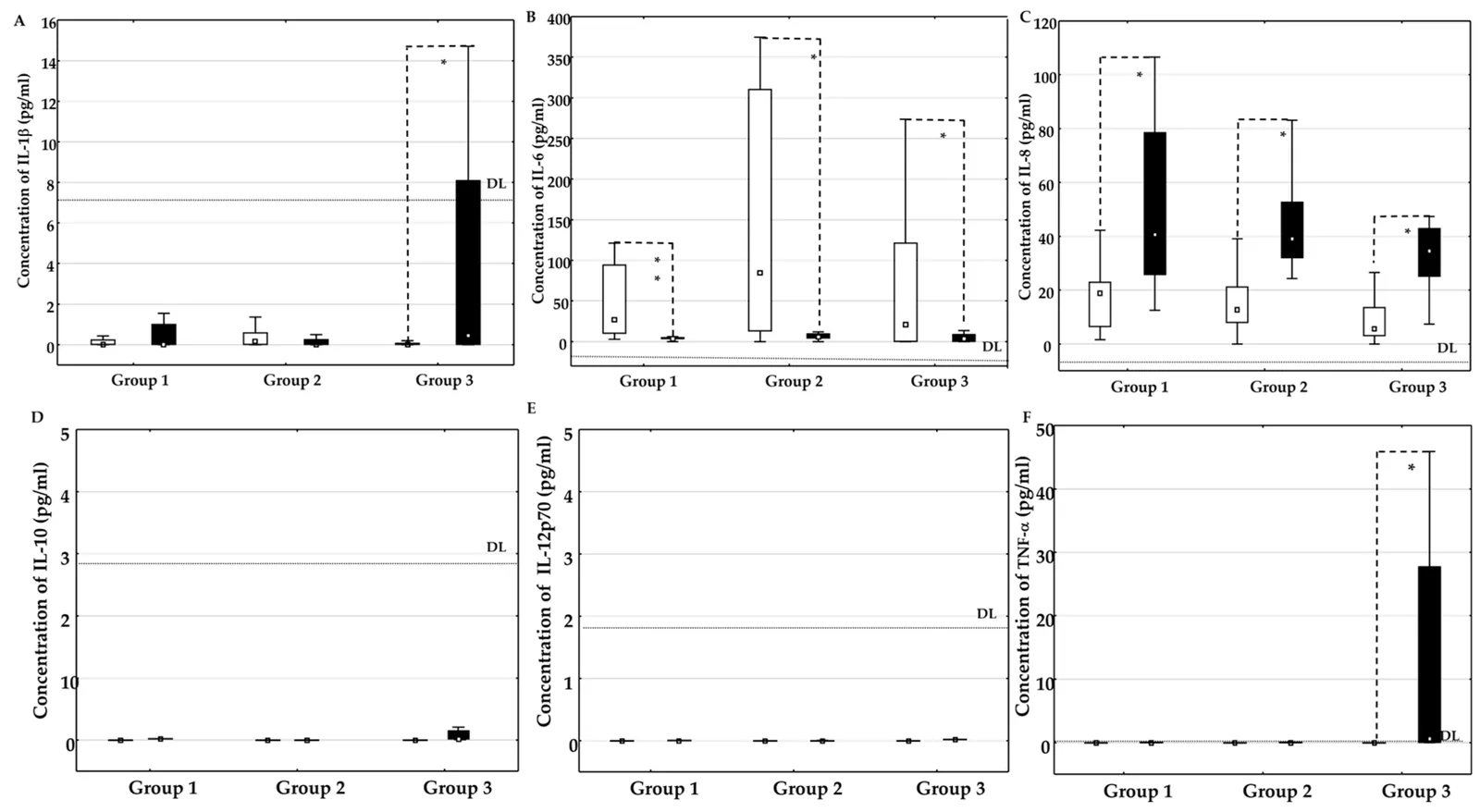
Comparison of IL-1β (**A**), IL-6 (**B**), IL-8 (**C**), IL-10 (**D**), IL-12p70 (**E**), and TNF-α (**F**) in aqueous humor (□) and serum (■) in Group 1, Group 2 and Group 3. * Level of statistical significance: *p* < 0.025. Data are expressed as median and 25th–75th percentile.

Figure 4 illustrates a comparison of IL-1β (A), IL-6 (B), IL-8 (C), IL-10 (D), IL-12p70 (E), and TNF-α (F) concentrations in the aqueous humor and serum of patients in Group 1, Group 2, and Group 3. Concentrations of IL-6 and IL-8 were detectable in aqueous humor and serum, whereas concentrations of IL-1β, IL-10, IL-12p70, and TNF-α were below the assay detection limit. IL-6 concentrations were statistically significantly higher in aqueous humor than in serum in all groups, while concentrations of IL-8 were statistically higher in serum than in aqueous humor in all groups.

### 3.5. Correlation Between Cytokine Concentrations in Aqueous Humor and Visual Acuity, Intraocular Pressure, Central Corneal Thickness, Endothelial Cell Density and Intraocular Lens Power

The correlations between aqueous humor concentrations of IL-6 and IL-8 and clinical parameters (VA, IOP, CCT, EDC and IOL) are presented in Table 2 and Table 3. A significant positive correlation between IL-6 concentration and IOL was observed only in Group 1 (Table 2).

### 3.6. Correlation Between Cytokine Concentrations in Serum and Visual Acuity, Intraocular Pressure, Central Corneal Thickness, Endothelial Cell Density and Intraocular Lens Power

The correlations between cytokine concentrations of IL-6 and IL-8 and clinical parameters (VA, IOP, CCT, ECD, and IOL) are presented in Table 4 and Table 5. A significant positive correlation was observed between IL-6 concentration and IOP, as well as between IL-8 concentration and IOP, in Group 3. No significant correlations were found in Group 1 or Group 2.

## 4. Discussion

This study investigated the effects of two commonly used antiglaucoma therapies, latanoprost and timolol–dorzolamide, on cytokine profiles in the aqueous humor and serum of patients with PEXG. The aim was to investigate the role of antiglaucoma therapy in modulating inflammatory cytokines involved in the pathobiology of PEXG. Although inflammation is increasingly recognized as a contributing factor in PEXG [30], the specific impact of antiglaucoma medications on these processes remains incompletely understood. Therefore, we evaluated the cytokine profile in the aqueous humor and serum of patients with PEXG undergoing cataract surgery and assessed its association with clinical parameters.

Our results showed that, among the analyzed cytokines, only IL-6 and IL-8 concentrations in aqueous humor exceeded the assay’s limit of detection. Although the concentrations of both cytokines were higher in Groups 1 and 2 than in Group 3, these differences did not reach statistical significance.

Zenkel et al. [26] investigated cytokine concentrations across different stages of PEXS and PEXG and compared them with cataract controls. They reported that, although most cytokines were comparable between groups, IL-6 and IL-8 levels were significantly elevated in the early stage of PEXS, whereas no significant differences were observed in later stages or in PEXG [26]. These findings are partially consistent with our results. In our study, IL-8 concentrations also showed a tendency to increase, with the highest values observed in the latanoprost-treated group and the lowest in controls; however, this difference did not reach statistical significance. Similarly, IL-6 levels demonstrated an increasing trend, with the highest concentrations observed in the timolol–dorzolamide group and the lowest in controls, although without statistical significance.

In contrast, other studies have demonstrated lower IL-6 levels in the aqueous humor of patients with PEXG and POAG compared with controls, highlighting the complexity and heterogeneity of cytokine responses in glaucomatous disease [35]. In normal-tension glaucoma (NTG), serum IL-6 concentrations are associated with disease severity. Although the difference did not reach statistical significance, concentrations were higher in patients with advanced NTG than in those with early or moderate stages of the disease [42]. Advanced-stage POAG patients exhibited significantly higher IOP and serum IL-6 concentrations than those in the early to moderate stage [43].

In our study, we found a positive correlation between serum concentrations of IL-6 and IL-8 and IOP in Group 3. This finding suggests that even within the physiological range, higher concentrations of these proinflammatory cytokines may be associated with higher IOP. However, since all participants were healthy and this was a correlational analysis, it is not possible to conclude that IL-6 or IL-8 directly caused an increase in IOP. It is more likely that the finding reflects a complex interaction between systemic immune activity and the mechanisms that regulate eye pressure.

IL-1β plays a critical role in retinal ganglion cell survival and is implicated not only in inflammatory and immune responses but also in neurodegenerative processes [44]. IL-1β activates immune cells and induces inflammation in ocular tissues. It disrupts the normal function and metabolism of trabecular meshwork cells, leading to increased resistance to aqueous humor outflow and elevated intraocular pressure [27]. This association supports the hypothesis that higher IL-1β levels may be linked to poorly controlled IOP, contributing to disease progression, structural damage, and irreversible vision loss. In our study, IL-1β concentrations in the aqueous humor were below the detection limit of the BD CBA method in most participants. A similar finding was reported by Zenkel et al. [26], who, using a multiplex bead immunoassay, also found that IL-1β concentrations in the aqueous humor were below the method’s sensitivity in patients with PEXS and PEXG. In contrast, the same study noted elevated IL-6 and IL-8 concentrations in the early stages of PEXS, suggesting that different cytokines may play distinct roles at various stages of the disease.

According to the instructions for the BD CBA Human Inflammatory Cytokines kit, the sensitivity for detecting IL-1β is lower than for the other cytokines tested. Consequently, it is possible that the 20 pg/mL standard point on the IL-1β curve does not yield a signal intensity above 0 pg/mL. Given these instructions, and since our results did not exceed the detection limit for this cytokine, we must consider, in drawing conclusions, that ultra-sensitive platforms may be required to detect low-grade changes in these specific cytokines.

Previous studies have investigated cytokine profiles in the aqueous humor across various forms of glaucoma, typically in the absence of significant comorbidities [31,35,37,45,46,47,48,49], and have demonstrated dysregulation of inflammatory mediators in PEXG, although the reported cytokine profiles are not entirely consistent. Increased IL-8 expression has been observed in both PEXS and PEXG [35,44,49], while TGF-β, a key regulator of extracellular matrix (ECM) remodeling, together with IL-8 and serum amyloid A (SAA)—an inflammatory marker produced by the liver under the influence of IL-1, IL-6, and TNF-α—has been implicated in IOP elevation [35]. Concentrations of TGF-β, IL-8, and SAA were significantly higher in aqueous humor samples from patients with POAG and PEXG [29]. A positive correlation was found between IOP, the number of glaucoma medications, and increased cytokine concentrations in the aqueous humor. It has been suggested that increased cytokine levels are more likely associated with elevated IOP than with the direct use of glaucoma medications [29]. It was proposed that oxidative stress induces cytokine production, which in turn promotes ECM remodeling within the trabecular meshwork, thereby increasing aqueous humor outflow resistance and contributing to elevated IOP [49]. IL-6 and IL-8 have been associated with altered inflammatory profiles in pseudophakic glaucomatous eyes [46], whereas lower levels of multiple cytokines have been reported in primary congenital glaucoma than in POAG [47]. Latanoprost treatment has been shown to significantly reduce aqueous humor TGF-β1 levels in patients with PEXG and is the only topical antiglaucoma therapy examined [37]. Although IL-6 is considered an important inflammatory mediator in glaucoma, decreased aqueous humor IL-6 concentrations have also been reported in both POAG and PEXG, highlighting the complexity of cytokine regulation and the potential influence of disease stage and treatment on cytokine expression [35].

Although the role of cytokines in ocular diseases, including PEXG, has been explored, data on their relationship with antiglaucoma therapy remains limited.

Chronic inflammatory reactions are characterized by the secretion of proinflammatory cytokines, including IL-1, IL-6, IL-8, and TNF-α [26], which are considered key factors in the onset and progression of glaucoma. Increased concentrations of inflammatory proteins and activation of inflammation-related pathways have been observed in different types of glaucoma. Inflammatory proteins enhance the secretion of IL-1, IL-6, and TNF-α [48,50,51]. These cytokines contribute to disease progression in PACG [30] and are associated with higher IOP values in the development of POAG [51]. Elevated concentrations of IL-1, IL-6, and TNF-α have also been found in patients with uveitis associated with Behçet’s disease, where high levels of C-reactive protein are linked to the inflammatory process in glaucoma [52].

Although the power of the intraocular lens depends on axial length and corneal curvature, we observed statistically significantly higher values in Group 2. However, this clinical parameter showed a statistically significant positive correlation with IL-6 levels in Group 1.

Interestingly, despite the well-documented proinflammatory potential of PGAs, IL-6 and IL-8 levels were not statistically different in Group 1 compared with Group 2. Konstas et al. [37] showed that latanoprost therapy reduces levels of TGF-β1 in the aqueous humor of patients with PEXG compared with the timolol-treated group. This finding may indicate that PGA-based therapy does not necessarily exacerbate intraocular inflammation at the cytokine level in a clinically meaningful way. Instead, it supports the notion that PGAs remain a safe and effective long-term treatment option, although their effects on subclinical inflammation require further investigation.

Reduced ECD observed before cataract surgery in patients with PEXG, together with the greater postoperative endothelial cell loss, suggests an inherent vulnerability of the corneal endothelium. A clinically significant decrease in ECD can lead to corneal decompensation, resulting in corneal clouding and reduced visual acuity in patients with PEXG [53]. Consistent with these findings, our results showed a reduction in ECD in patients with PEXG. However, despite the observed trends, our study did not demonstrate statistically significant differences in clinical parameters such as ECD between treatment groups. This suggests that topical antiglaucoma therapy may not have a measurable effect on ECD and that endothelial changes in PEXG are more likely related to the underlying disease process rather than medication effects.

Reduced CCT has been identified as an important risk factor for glaucoma development, as highlighted by the Ocular Hypertension Treatment Study, which showed that thinner corneas are associated with an increased risk of progression to POAG [54]. By extension, patients with PEXS and lower CCT may also be at higher risk of developing or progressing to PEXG [37]. Our results showed decreased CCT in the latanoprost-treated group.

PGAs are widely established as first-line therapies for reducing IOP because of their efficacy in enhancing uveoscleral outflow; however, their role in inflammatory signaling remains clinically relevant. Latanoprost, a prostaglandin F2α analog, acts through eicosanoid pathways that are intrinsically linked to inflammatory mediator cascades, which may contribute to ocular surface and intraocular inflammatory responses [55,56].

Disruption of the blood–aqueous barrier is a key mechanism underlying these effects, allowing proteins and inflammatory cells to enter the anterior chamber. This phenomenon is particularly concerning in postoperative eyes, where barrier integrity is already compromised after anterior segment surgery. Recent evidence suggests that PGAs may modulate intraocular immune responses and lipid mediator profiles, further supporting their ability to influence inflammatory pathways within the eye [56]. Additionally, emerging clinical data indicates a potential association between latanoprost use and an increased risk of postoperative complications with an inflammatory component, such as graft rejection after corneal transplantation, highlighting the immunomodulatory effects of these agents [57]. Although the incidence of clinically significant inflammation remains relatively low, cases of anterior chamber reactions characterized by the presence of inflammatory cells have been documented in patients receiving latanoprost therapy, particularly in susceptible individuals [55]. Therefore, while PGAs remain highly effective and generally well tolerated, careful consideration is warranted when prescribing these agents in the immediate postoperative period or in eyes predisposed to inflammation.

One of the most well-characterized attributes of an aging immune system is an aberrant, chronic, low-grade proinflammatory state, thought to occur to a greater extent in females than in males [58]. In women, menopause is characterized by a sharp and permanent reduction in ovarian estrogen production, accompanied by a more gradual decline in androgen levels. In contrast, men experience a progressive age-related decline in androgen production, particularly testosterone, but circulating testosterone concentrations generally remain several-fold higher than those observed in women throughout later life [59].

The decline in estradiol after menopause is associated with a shift toward a pro-inflammatory immune profile, characterized by increased production of IL-1, IL-6, and TNF-α, along with reduced production of IL-10. Similarly, androgen deficiency in men has been associated with increased production of IL-1 and TNF-α and decreased IL-10, although this relationship is less consistent and is not observed in all aging men [60].

This research has several limitations. The number of aqueous humor samples analyzed was restricted by the small volume that can be aspirated from the eye, and the procedure cannot be repeated, preventing the monitoring of dynamic changes over time. The sample size was relatively small, limiting the statistical power to detect more subtle differences, and the study included only patients from a single ophthalmology clinic. In addition, the study lacked a true untreated control group, which was not feasible because patients with PEXG require continuous medical therapy.

In our study, the high proportion of female participants in Group 2 may limit the applicability of the findings across sexes. Incorporating additional inflammatory mediators alongside comprehensive markers would provide a more complete understanding of the pathophysiological mechanisms involved in PEXG. Since this study was not designed to examine the impact of sex on disease development or progression, it is not possible to say with certainty whether this difference reflects true biological differences or is partly due to the composition of the sample studied.

Including multiple centers from different countries would provide better insight into the impact of antiglaucoma therapy on cytokine concentrations in the aqueous humor, as well as the relationship between medications and clinical signs of inflammation and their influence on surgical outcomes.

## 5. Conclusions

This study provides evidence that antiglaucoma therapy may influence inflammatory cytokine profiles in the aqueous humor of patients with PEXG.

Although IL-6 and IL-8 cytokines showed trends toward altered concentrations, these differences did not reach statistical significance, indicating that inflammatory changes in PEXG may be subtle and influenced by multiple factors such as disease stage and treatment modality. Importantly, the lack of significant differences in ECD between treatment groups suggests that corneal endothelial alterations are more likely related to the underlying disease process rather than the effects of topical therapy. Taken together, these findings support the presence of a low-grade inflammatory milieu in PEXG while PGAs remain safe and effective first-line therapy.

## Figures and Tables

**Table 1:** Comparison of demographic and clinical characteristics of the patients.

Characteristics	Group 1	Group 2	Group 3	*p* Value
*n*	20	20	20	
Sex (male/female)	15/5	4/16	9/11	**0.002**
Age (years; age range)	78 (66–87)	82 (68–92)	79 (64–86)	0.154
VA OD	0.30 (0.09–0.40)	0.13 (0.05–0.35)	0.25 (0.13–0.45)	0.639
VA OS	0.25 (0.08–0.50)	0.30 (0.15–0.40)	0.20 (0.10–0.30)	0.674
VA	0.32 (0.08–0.45)	0.29 (0.14–0.44)	0.29 (0.16–0.48)	0.935
IOP OC DEX (mmHg)	18 (16–20)	17 (16–19)	16 (15–17)	0.096
IOP OC SIN (mmHg)	18 (17–20)	17 (16–19)	17 (16–17)	0.255
IOP (mmHg)	17.25 (17–19.75)	17.25 (16–18.25)	17 (15.75–17)	0.053
CCT (mm)	520 (508–538)	551 (525–561)	551 (517–566)	**0.014**
ECD (cell/mm^2^)	2449 (2274–2671)	2572 (2355–2653)	2557 (2426–2725)	0.652
IOL (D)	20 (19–21.5)	21.5 (21–22.8)	19 (17.5–21.5)	**0.015**

Continuous variables are presented as median and 25th–75th percentiles, and categorical data are presented as number of cases. Group 1—patients on latanoprost therapy, Group 2—patients with timolol and dorzolamide therapy, Group 3—control group without PEXG and antiglaucoma therapy. VA OD—visual acuity right eye, VA OS—visual acuity left eye, VA—visual acuity, IOP OC DEX—intraocular pressure right eye, IOP OC SIN—intraocular pressure left eye, IOP—intraocular pressure, CCT—central corneal thickness, ECD—endothelial cell count, IOL—intraocular lens.

**Table 2:** Correlation between the concentration of IL-6 in aqueous humor and VA; IOP, CCT, EDC, IOL in Group1, Group 2 and Group 3.

Correlation	Concentration of IL-6 (pg/mL)					
	Group 1		Group 2		Group 3	
	*r*	*p*	*r*	*p*	*r*	*p*
VA	−0.199	0.399	−0.040	0.867	−0.185	0.434
IOP (mmHg)	−0.230	0.328	−0.442	0.051	0.370	0.108
CCT (mm)	−0.313	0.178	0.303	0.194	−0.121	0.610
ECD (cell/mm^2^)	0.029	0.901	0.372	0.106	−0.420	0.065
IOL (D)	**0.489**	**0.028**	−0.040	0.867	−0.037	0.877

Group 1—patients on latanoprost therapy, Group 2—patients with timolol and dorzolamide therapy, Group 3—control group without PEXG and antiglaucoma therapy. VA—visual acuity, IOP—intraocular pressure, CCT—central corneal thickness, ECD—endothelial cell count, IOL—intraocular lens. *p*-level of statistical significance, r—correlation coefficient.

**Table 3:** Correlation between the concentration of IL-8 in aqueous humor and VA; IOP, CCT, EDC, IOL in Group1, Group 2 and Group 3.

Correlation	Concentration of IL-8 (pg/mL)					
	Group 1		Group 2		Group 3	
	*r*	*p*	*r*	*p*	*r*	*p*
VA	−0.413	0.070	0.010	0.966	0.102	0.668
IOP (mmHg)	−0.027	0.908	0.320	0.568	0.197	0.404
CCT (mm)	−0.328	0.157	−0.184	0.436	0.321	0.167
ECD (cell/mm^2^)	−0.348	0.132	0.092	0.699	−0.235	0.317
IOL (D)	0.130	0.582	0.113	0.635	−0.264	0.261

Group 1—patients on latanoprost therapy, Group 2—patients with timolol and dorzolamide therapy, Group 3—control group without PEXG and antiglaucoma therapy. VA—visual acuity, IOP—intraocular pressure, CCT—central corneal thickness, ECD—endothelial cell count, IOL—intraocular lens. *p*-level of statistical significance, r—correlation coefficient.

**Table 4:** Correlation between the concentration of IL-6 in serum and VA, IOP, CCT, EDC, and IOL in Group 1, Group 2 and Group 3.

Correlation	Concentration of IL-6 (pg/mL)					
	Group 1		Group 2		Group 3	
	*r*	*p*	*r*	*p*	*r*	*p*
VA	−0.332	0.152	0.115	0.629	−0.260	0.268
IOP (mmHg)	0.171	0.470	0.358	0.881	**0.536**	**0.015**
CCT (mm)	−0.186	0.430	0.357	0.121	0.797	0.738
ECD (cell/mm^2^)	−0.008	0.971	−0.124	0.601	0.407	0.074
IOL (D)	0.007	0.975	0.037	0.875	0.052	0.827

Group 1—patients on latanoprost therapy, Group 2—patients with timolol and dorzolamide therapy, Group 3—control group without PEXG and antiglaucoma therapy. VA—visual acuity, IOP—intraocular pressure, CCT—central corneal thickness, ECD—endothelial cell count, IOL—intraocular lens. *p*-level of statistical significance, r—correlation coefficient.

**Table 5:** Correlation between the concentration of IL-8 in aqueous humor and VA; IOP, CCT, EDC, IOL in Group1, Group 2 and Group 3.

Correlation	Concentration of IL-8 (pg/mL)					
	Group 1		Group 2		Group 3	
	*r*	*p*	*r*	*p*	*r*	*p*
VA	−0.215	0.362	0.136	0.567	−0.183	0.438
IOP (mmHg)	0.360	0.118	0.099	0.677	**0.609**	**0.004**
CCT (mm)	−0.350	0.130	−0.132	0.576	0.023	0.923
ECD (cell/mm^2^)	−0.002	0.991	0.181	0.444	0.227	0.336
IOL (D)	0.188	0.427	0.0623	0.794	−0.055	0.816

Group 1—patients on latanoprost therapy, Group 2—patients with timolol and dorzolamide therapy, Group 3—control group without PEXG and antiglaucoma therapy. VA—visual acuity, IOP—intraocular pressure, CCT—central corneal thickness, ECD—endothelial cell count, IOL—intraocular lens. *p*-level of statistical significance, r—correlation coefficient.

## Data Availability

The data presented in this study are available on request.

## Abbreviations

The following abbreviations are used in this manuscript:

APC	allophycocyanin
AUC	area under the curve
CBA	cytometric bead array
CCT	central corneal thickness
CME	cystoid macular edema
ECD	endothelial cell density
IFN	interferon gamma
IL	interleukin
IOL	intraocular lens
IOP	intraocular pressure
NTG	normotensive glaucoma
PE	phycoerythrin
PEX	pseudoexfoliation
PEXG	pseudoexfoliation glaucoma
PEXS	pseudoexfoliation syndrome
PGA	prostaglandin analogues
POAG	primary open angle glaucoma
RGC	retinal ganglion cell
SAA	serum amyloid A
TGFβ	transforming growth factor beta
TNF	tumor necrosis factor
VA	visual acuity
